# Supplementary material for: Expression Quantitative Trait Locus Study of Non-Syndromic Cleft Lip with or without Cleft Palate GWAS Variants in Lip Tissues
Source: Cells. 2022 Oct 18;11(20):3281. doi: 10.3390/cells11203281 (PMC9600070; doi:10.3390/cells11203281)
Supplement: Supplementary file 1 [file cells-11-03281-s001.zip › TableS1&TableS3&TableS5.pdf]

Table S1. Demographics of the Study Cohort

Demographic variable	Ratio/ Mean (SD)
Male: Female	22:18
Age (years)	1.95 (2.60)
NSCLO: NSCLP	16:24

Note: The study cohort consisted of 40 non-syndromic cleft lip with or without cleft palate. Data were shown as ratio or mean (SD). **NSCLO:** non-syndromic cleft lip only; **NSCLP:** non-syndromic cleft lip and cleft palate; **SD:** standard deviation.

Table S3. Gene Ontology Pathway Enrichment Analysis on eGenes at Biological Process Level

#Term	ID	Input	Total	p-value	FDR	-log (p value)
G protein-coupled receptor signaling pathway	GO:0007186	36	1132	1.70E-08	1.78E-05	7.77
keratinization	GO:0031424	13	175	1.37E-07	7.40E-05	6.86
olfactory receptor activity	GO:0004984	17	427	7.19E-06	1.28E-03	5.14
detection of chemical stimulus involved in sensory perception of smell	GO:0050911	17	427	7.19E-06	1.28E-03	5.14
G protein-coupled receptor activity	GO:0004930	22	739	2.89E-05	4.31E-03	4.54
cellular response to tumor necrosis factor	GO:0071356	8	125	9.61E-05	1.25E-02	4.02
positive regulation of transcription initiation from RNA polymerase II promoter	GO:0060261	4	20	1.14E-04	1.25E-02	3.94
chemical synaptic transmission	GO:0007268	11	248	1.20E-04	1.25E-02	3.92
positive regulation of GTPase activity	GO:0043547	12	307	1.85E-04	1.61E-02	3.73
regulation of transcription by RNA polymerase II	GO:0006357	20	751	2.77E-04	2.06E-02	3.56
signal transduction	GO:0007165	24	1013	3.76E-04	2.62E-02	3.42
regulation of synaptic transmission, glutamatergic	GO:0051966	4	30	4.59E-04	2.90E-02	3.34

Table S5. eSNP-TF-eGene regulatory relationships

risk-eSNP	TF	eGene
rs4887163	CHD1	*CSK, UBL7*
rs12595017	GATA6	*UBL7*
rs35963929	GATA6	*RNA5SP484*
rs11698548	GATA6	*RNA5SP484*
rs11698990	GATA6	*RNA5SP484*
rs1409904	GATA6	*SPRY2*
rs8037714	KDM4A	*CSK, UBL7*
rs7166656	CTCF	*UBL7*
rs1821847	CTCF	*UBL7*
rs8027066	CTCF	*CSK, UBL7*
rs12441932	CTCF	*SCAMP2, CSK, UBL7*
rs1821847	TBP	*UBL7*

eSNP: eQTL variant; **TF**: transcription factor; **eGene**: eQTL gene.
